# Hypochlorous Acid: Clinical Insights and Experience in Dermatology, Surgery, Dentistry, Ophthalmology, Rhinology, and Other Specialties

**DOI:** 10.3390/biomedicines13122921

**Published:** 2025-11-28

**Authors:** Vanda Haralović, Mislav Mokos, Sanja Špoljar, Lorena Dolački, Mirna Šitum, Liborija Lugović-Mihić

**Affiliations:** 1 Department of Dermatovenereology, University Hospital Center Sestre Milosrdnice, Vinogradska cesta 29, 10000 Zagreb, Croatia; vanda.haralovic@gmail.com (V.H.);; 2 School of Dental Medicine, University of Zagreb, Gundulićeva 5, 10000 Zagreb, Croatia

**Keywords:** topical treatment, antimicrobial therapy, anti-inflammatory drugs, immunomodulator, healing, ulcer, atopic dermatitis, surgical procedures, postoperative care, disinfectant

## Abstract

**Background:** Hypochlorous acid (HOCl) is an integral component of the human innate immune system. It possesses antimicrobial properties and is available in solution, dermal spray, and scar gel forms. **Objectives/Methods:** This review presents data from studies on the clinical use of HOCl in various specialties, including dermatology, surgery, dentistry, ophthalmology, and rhinology. **Results:** Due to its anti-inflammatory/antimicrobial/immunomodulatory and healing properties, HOCl is advantageous in treating various skin disorders: ulcus cruris (and wound care), diabetic ulcers, atopic dermatitis, seborrheic dermatitis, pruritus, acne vulgaris, etc. Also, the application of a HOCl spray/gel after surgical procedures may prevent infection, reduce inflammation, and accelerate healing. HOCl is also effective and safe for the prevention and treatment of hypertrophic and keloid scars. Growing evidence shows a broader role for HOCl in limiting cancer cell survival and slowing tumor growth. It is also important in treating various viral infections like SARS-CoV-2 (coronavirus), influenza, and herpes, thereby helping to prevent the spread of aerosols. In addition, since HOCl is an endogenous compound naturally present in mammals with a high safety profile, it may be an effective bacterial disinfectant in dental waterlines. In ophthalmology, adjuvant treatment with HOCl ophthalmic spray can reduce the duration of antibiotic/corticosteroid use, even in severe blepharitis. To fully harness the protective/therapeutic properties of HOCl, future advancements will rely on the development of new chemical compounds and sophisticated pharmaceutical formulations. **Conclusions:** The majority of clinical studies have confirmed that HOC1 is useful in therapy, although the results are not entirely consistent. Further research is essential to optimize HOCl dosing and to develop controlled-release systems aimed at maximizing its anti-inflammatory and photoprotective effects while minimizing tissue irritation and damage.

## 1. Introduction

**Figure 1:**
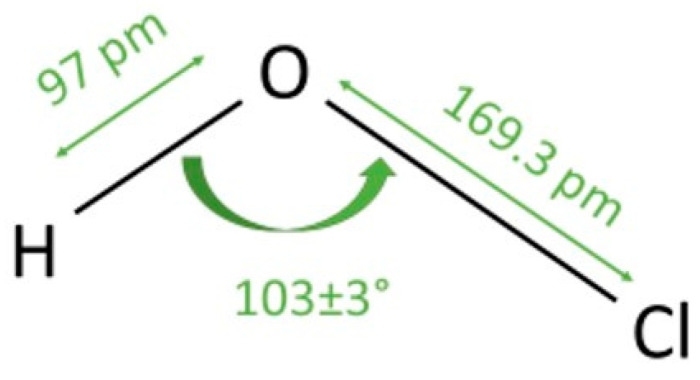
Chemical structure of the molecule of HOCl (this figure was created by the authors, based on reference [2]).

Hypochlorous acid (HOCl) is a small, highly reactive oxychlorine species that plays an important role in the innate immune system. It was first described in the early nineteenth century and later identified as a product of myeloperoxidase activity in neutrophils. HOCl is important in the oxidative burst, since it contributes to the rapid destruction of bacteria, fungi, and viruses [1]. Chemically, HOCl is a weak acid formed by the dissolution of chlorine in water, existing in equilibrium with its conjugate base, hypochlorite (OCl^−^) (Figure 1) [2]. The structure of OCl^−^ consists of a simple chlorine–oxygen bond. However, the reactivity of HOCl is diverse and biologically significant. HOCl functions as a strong oxidizing and chlorinating agent. It commonly reacts with sulfhydryl groups, amines, and nucleotides, and, therefore, inactivates microbial enzymes, disrupts membranes, and damages nucleic acids [3,4]. These reactions result in rapid pathogen inactivation, and host tissues are relatively spared due to endogenous antioxidant defenses. HOCl also modulates inflammatory signaling, reduces biofilm formation, and promotes wound healing by supporting re-epithelialization and angiogenesis [5]. Both in vitro and in vivo studies have confirmed the antimicrobial properties of HOCl. Namely, this molecule has a broad spectrum of activity and minimal risk of resistance development [6,7].

HOCl is effective for pathogen elimination. However, its strong oxidizing capacity also places significant demands on endogenous antioxidant systems. Oxidative stress is a well-recognized contributor to tissue injury, and antioxidants act as crucial modulators that neutralize reactive species and maintain redox homeostasis [8]. Numerous small-molecule antioxidants, both natural and synthetic, exert protective effects by scavenging free radicals, as demonstrated by structurally diverse compounds with potent antioxidant capacity in biochemical assays. Importantly, HOCl can react rapidly with phenolic groups, thiols, and other electron-donating moieties, thereby suppressing the antioxidant potential of these compounds. Besides HOCl being indispensable for host defense, its high reactivity can transiently overwhelm local antioxidant reserves. Understanding this balance is essential for interpreting HOCl’s physiological actions as well as its therapeutic safety profile in clinical formulations [8].

HOCl has gradually been introduced into everyday clinical practice. Early studies have focused on its disinfectant potential. However, advances in electrochemical technology enabled the generation of stable, pH-controlled formulations that are suitable for medical use. Over the last two decades, these innovations have enabled the dermatologic use of HOCl for wound cleansing, burn care, and the treatment of chronic ulcers. More recently, HOCl use has extended to ophthalmology, dentistry, and rhinology [9,10,11,12,13,14]. Numerous clinical studies and systematic reviews have reported that HOCl has good tolerability, and that it is not cytotoxic, while being effective in reducing microbial burden and inflammation [15,16]. Given its natural role in host defense, favorable safety profile, and growing body of clinical evidence, HOCl represents a unique example of a molecule that bridges innate immunity and therapeutic application.

A clear understanding of the basic chemical behavior of HOCl is essential for interpreting its diverse biological effects. Although its underlying reactions are detailed throughout the manuscript, it is important to emphasize here that the biological activity of HOCl arises directly from its simple yet highly reactive structure [17]. As a small oxychlorine molecule capable of existing in a pH-dependent equilibrium with OCl^−^, HOCl acts as a potent oxidant and chlorinating agent, selectively modifying sulfur- and nitrogen-containing residues on biomolecules [18]. These reactions form the mechanistic foundation for all of HOCl’s bioactive roles (including antimicrobial activity, modulation of inflammatory signaling, and interactions with host antioxidants). This chemical reactivity explains both its efficiency as an innate defense molecule and the need for careful control when used therapeutically [4].

The purpose of this narrative review is to provide an updated synthesis of current knowledge on the use of HOCl in clinical practice in various medical disciplines. For this purpose, we first conducted an analysis of articles published so far based on clinical studies with clinical experiences with HOCl and similar preparations, with the aim of presenting them in tables (given that this is a narrative article, while we did not conduct a meta-analysis). For this purpose, we analyzed the PubMed database in September 2025 and used the keywords “hypochlorous acid” and “treatment” and “clinical study”. We considered and collected only articles that were research/clinical studies published from 2000 onward, while we excluded reviews, cases, websites, books, letters to the editor and other data, as well as in vitro research and studies older than 2000 or some studies that did not directly address the effect of HOCl. After searching PubMed and other prominent medical databases, we processed data obtained from 61 original clinical studies, all published since 2000, which served to present the results by clinical branch in tables. Thus, we wanted to provide an overview that provides information on the clinical application of HOCl, the biological mechanisms and the expansion of clinical application in dermatology, wound care, dentistry, ophthalmology, rhinology and other relevant fields. Then, in a second step, to provide a broader overview of this topic and to further interpret the results of published clinical studies, we reviewed reviews, case reports, medical websites, books, letters to the editor, and others, which we used to provide a broader insight into this topic in this narrative review article. Finally, this review provides the chemistry behind, biological mechanisms of, and expanding clinical applications of HOCl across dermatology, wound care, dentistry, ophthalmology, rhinology, and other relevant fields.

## 2. Key Characteristics of Hypochlorous Acid Related to Its Use—Mechanistic Considerations: Immunomodulation and Antiviral Activity; pH, and the Effect of Antimicrobial Activity; Practical Use Parameters and Comparative Effectiveness; Suggested Dosing Guidance

At the molecular level, HOCl and its secondary chloramines display selective, rapid reactions with sulfur-containing residues (cysteine, methionine) and certain amines, generating reversible redox modifications that can function as “switches” on signaling proteins. This chemistry under physiological conditions is well characterized and underpins both antimicrobial effects and host-directed immunomodulation [18,19]. Endogenously, HOCl formed by myeloperoxidase reacts with taurine to yield taurine chloramine (TauCl/N-chlorotaurine), a longer-lived, less aggressive oxidant that down-regulates inflammatory signaling. TauCl inhibits NF-κB activation with concomitant suppression of inducible nitric-oxide synthase and tumor necrosis factor-alpha (TNF-α) expression in activated macrophages, and it promotes pro-resolution efferocytosis via NRF2–HO-1 pathway engagement; these effects are summarized in contemporary reviews of HOCl biology [4,20].

For viruses, purified HOCl produces oxidative damage to key structural proteins and lipids of the virion. High-purity HOCl causes irreversible viral protein aggregation and disrupts binding of the SARS-CoV-2 spike to ACE2, providing a direct biochemical explanation for the rapid loss of infectivity observed in experimental systems [21]. Consistent with these mechanistic observations, HOCl solutions inactivate coronaviruses (including SARS-CoV-2) and other enveloped viruses under laboratory conditions [22]. Beyond exogenous application, epithelial and other non-myeloid cells can mount an intracellular HOCl-dependent antiviral response: increasing intracellular chloride augments peroxidase-mediated HOCl production and suppresses replication of both enveloped and non-enveloped DNA/RNA viruses in vitro [23]. Finally, the endogenous chloramine derivative N-chlorotaurine exhibits broad activity against respiratory viruses (including coronaviruses and influenza) and inactivates enveloped viruses by oxidizing S-H and N-H groups on surface and capsid proteins, supporting a coherent mechanistic framework that links HOCl biochemistry to antiviral effects across matrices [24].

Concerning HOCl-related speciation, pH, and its effect on antimicrobial activity, there are a few important things to consider: the antimicrobial efficacy of chlorine oxidants is governed by the acid–base equilibrium between HOCl and its conjugate base, hypochlorite (OCl^−^). HOCl is a weak acid with pK_a ≈ 7.4–7.5 at 25 °C; consequently, HOCl predominates in mildly acidic to neutral solutions, whereas OCl^−^ becomes dominant at alkaline pH. Under very acidic conditions (pH < ~4), molecular chlorine (Cl_2_) can form; at alkaline pH (>~8), the equilibrium shifts toward OCl^−^ [25]. This speciation matters because HOCl is both more membrane-permeant (electrically neutral) and more strongly oxidizing in physiological media than OCl^−^, enabling faster penetration of microbial envelopes and more rapid oxidation/chlorination of cysteine-, methionine-, and amine-containing targets. Comparative studies and mechanistic reviews consistently identify HOCl as the principal germicidal species, with substantially greater bactericidal activity than OCl^−^ at equivalent free available chlorine (FAC) [26,27]. Practically, maximal antimicrobial performance is observed when the formulation maintains a high fraction of HOCl (roughly pH ~5–6.5), while very alkaline solutions—despite identical FAC—show reduced activity because OCl^−^ predominates and penetrates cells poorly. This pH–efficacy coupling has been demonstrated across bacteria and spores and underpins the superior performance of slightly acidic electrolyzed/“superoxidized” waters versus hypochlorite solutions buffered alkaline [28,29]. Together, these chemical principles explain clinical observations: formulations engineered to stabilize HOCl in the mildly acidic–neutral range deliver stronger and faster antimicrobial effects at lower nominal chlorine levels, while also avoiding chlorine gas formation at very low pH and the decreased efficacy associated with alkaline hypochlorite solutions.

It is needed to mention HOCl’ practical use parameters and comparative effectiveness, as well as the suggested dosing guidance (by setting). Thus, the effective concentration and contact time of HOCl depend on the intended clinical use and matrix (skin, mucosa, ocular surface, or dental waterline), as well as on formulation pH and stability. Regulatory and health-technology assessments note that wound-care formulations cleared in the United States typically contain 100–200 ppm available (titrable) chlorine as HOCl, with comparable limits in European Class III products; these ranges balance antimicrobial potency with tissue tolerability in pH-neutral or slightly acidic stabilized solutions [28,30,31].

HOCl is especially suitable for skin and wound antisepsis (acute and chronic wounds). In clinical wound care, irrigation and short “soak” applications within the 100–200 ppm range are used at each dressing change, with contact times of several minutes to reduce bioburden before definitive coverage [32,33,34,35,36]. Randomized studies in diabetic foot and postoperative lesions found super-oxidized, pH-neutral HOCl solutions superior or at least as effective as standard local antiseptics (e.g., povidone-iodine), with faster resolution of infection and improved healing trajectories; these trials operationalized HOCl as a wound irrigant and topical adjunct within routine debridement and moist-wound protocols. Volunteer and early patient studies with “stabilized” HOCl further support protocolized pre-closure cleansing and peri-procedural use to reduce bacterial bioburden without cytotoxic injury to healing tissue. Additional perioperative reports describe lavage of the peritoneal cavity at approximately 100 ppm and external wound washing at approximately 200 ppm without safety signals, consistent with the above wound-care range [32,33].

HOCl is also useful in dentistry and for is effective on aerosol-borne viruses. In dental settings HOC1 is used in pre-procedural rinses, and continuous irrigation with HOCl in the handpiece water stream and/or brief pre-procedural rinses are used to mitigate aerosol transmission. An oral-aerosol simulation platform demonstrated complete inactivation of betacoronavirus OC43 (surrogate for SARS-CoV-2), influenza H1N1, and HSV-1 within 30 s at 100 ppm, supporting 30–60 s contact times for virucidal applications in the oral cavity. Complementary work shows that even 45–60 ppm can inactivate common oral pathogens and a coronavirus surrogate after transit through dental unit waterlines, indicating that low-tens of ppm are sufficient for continuous waterline delivery when contact is sustained by irrigation. Clinically, a randomized, controlled non-inferiority trial in postsurgical periodontitis found an HOCl antiplaque protocol non-inferior to chlorhexidine (CHX) for reducing plaque indices and pathogenic recolonization over 90 days, providing a comparator framework in which HOCl achieves outcomes similar to CHX without CHX-associated drawbacks (staining, dysgeusia).

In dentistry, HOCl at 100 ppm delivers rapid virucidal activity under aerosolized, saliva-rich conditions that challenge other common agents, allowing for real-world mitigation in operatories. For dental aerosol control, it is recommended to use 100 ppm HOCl, where a rapid virucidal effect is needed within 30–60 s. For continuous waterline irrigation where exposure is prolonged by ongoing spray, 45–60 ppm is recommended. Both approaches have experimental support under saliva-containing conditions [36].

HOCl may also be used in ophthalmology, primarily for eyelid hygiene and adjunctive therapy. On the periocular skin and lid margins, 0.01% HOCl (≈100 ppm) applied in sprays, wipes, or via ultrasonic atomization is used once to several times daily for blepharitis/meibomian gland dysfunction. For eyelid hygiene, it is reccommended to apply a 0.01% HOCl solution to lids/lashes (spray/wipes or atomized) once to several times daily, as tolerated; randomized data support conducting atomization with a 0.01% solution over approximately two weeks, with practice patterns commonly extending usage for maintenance [34]. Suggested dosing guidance (by setting), for wound bed preparation and dressing changes recommends irrigating or soaking the wound with a stabilized HOCl solution at 100–200 ppm for several minutes (commonly 3–10 min) before applying the final dressing (this process should be repeated with each change). This aligns with regulatory ranges and randomized wound studies and is consistent with protocols that prioritize bioburden reduction without impairing re-epithelialization [35]. In the treatment of infectious keratitis for adjunctive ophthalmic therapy, 0.01% HOCl eye drops have been used in combination with standard therapy [37].

The comparative effectiveness and safety of HOC1 is important to consider. Across wound-care indications, HOCl has demonstrated superiority over povidone-iodine in some randomized and quasi-experimental trials of diabetic foot and postsurgical lesions, and is as effective as chlorhexidine in periodontal postsurgical care, while maintaining a favorable tolerability profile on skin and mucosa [38].

Taken together, these data provide actionable dosing parameters and contact times for the principal clinical contexts in which HOCl is currently used, while situating HOCl’s performance against standard comparators where such evidence is available.

## 3. Hypochlorous Acid in Wound Care, Scar Management, and Postoperative Treatment

**Figure 2:**
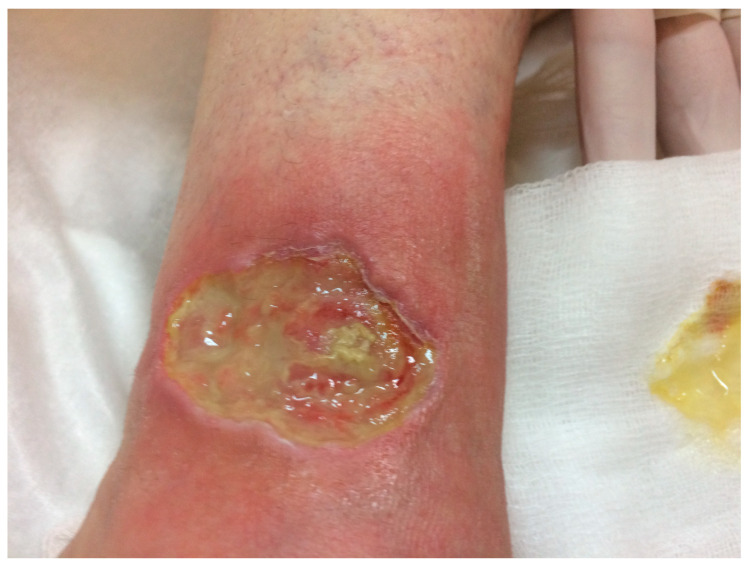
Chronic ulcer (the picture was taken/collected by the authors with patient consent).

In the wound healing process following procedures, there is often concern about pre- and post-operative wound care, as well as scar treatment (Figure 2) [5]. Physicians aim to balance antiseptic properties and potential cytotoxicity with the severity of bacterial colonization to ensure optimal healing conditions and improve treatment outcomes. It is useful to know that the immune system naturally produces HOCl, a compound that effectively eliminates bacteria and other invasive pathogens in the body, and exhibits antipruritic and anti-inflammatory effects [25,27,38]. HOCl solution has been extensively studied for the treatment of complex wounds, including diabetic foot ulcers, venous leg ulcers, sternal wounds following coronary bypass surgery, and postoperative treatment of diabetic foot ulcers [39]. HOCl is used topically for wounds due to its antimicrobial, anti-inflammatory, immunomodulatory properties, and its ability to promote wound healing [7,9,14,40,41,42].

According to research, HOCl effectively reduces bacterial counts in open wounds [43]. When HOCl irrigation solution was applied using an ultrasonic system, bacterial load was reduced by 4 to 6 log units. By the time the wounds closed, bacterial levels in the control group treated with saline increased to 10^5^, whereas in the HOCl-treated group, they remained at ≤10^2^. Regarding healing outcomes, unsuccessful postoperative wound closure was observed in over 80% of patients in the saline group, compared to only 25% in the HOCl group [43]. There may also be an effect on intraperitoneal wound care: in a clinical study assessing HOCl for intraperitoneal wound management, patients benefited from peritoneal cavity irrigation with 100 ppm HOCl, followed by wound rinsing with 200 ppm, without any adverse effects reported.

HOCl-containing spray or superoxidized solution is used for cleaning, irrigating, and disinfecting surgical wounds, which can reduce infection risk and improve healing outcomes [27,41,44,45,46,47]. The spray is applied to prepare the surgical site and disinfect before closure, and used twice daily for one week postoperatively to support healing. A transparent gel formulation is also available, combining modified silicone oil with HOCl for scar treatment (Celacyn), which may be used along with the dermal spray to improve scar appearance and manage associated itch and pain. HOCl gel is applied twice daily for 6 to 8 weeks. Neutral pH HOCl solution is considered a safe and effective biocide for skin and wound disinfection [46,47].

The relevance of HOCL extends beyond its use as a topical wound antiseptic and is increasingly being recognized at the global health policy level [30,31]. HOCl has proven itself to be useful not just as a topical wound antiseptic. Namely, a recent proposal submitted to the World Health Organization advocates for the inclusion of HOCl in the Model List of Essential Medicines for disinfection, antisepsis, and wound management. This initiative is based on various studies that prove the broad-spectrum antimicrobial efficacy, safety, and favorable tolerability of HOCl compared with conventional agents. In the area of disinfection, HOCl has been shown to inactivate a wide range of pathogens, including viruses, bacteria, fungi, and even resistant organisms such as human papillomavirus, for which many standard disinfectants remain inadequate [27,41,44,48]. Moreover, studies report reductions in microbial burden, faster readiness for closure, and improved patient comfort, with lower cytotoxicity than traditional antiseptics such as povidone-iodine or chlorhexidine. In addition, HOCl is increasingly utilized in medical device reprocessing and in emergency and humanitarian contexts. The reason for this lies in the stability, ease of production, and lack of toxic by-products of HOCl, which makes it particularly valuable in low-resource environments.

In the context of postoperative wound care and scar prevention, it is estimated that in developed countries, about 100 million people develop scars after elective or traumatic surgery, and approximately 15% of patients develop hypertrophic scars [49,50,51,52,53,54]. Scars may also be accompanied by itching, stiffness, contracture, sensitivity, and pain [51]. Aesthetically unacceptable scars can lead to psychosocial consequences such as reduced self-esteem, stigmatization, difficulty with daily activities, anxiety, and depression [52]. A hypertrophic scar remains within the original wound boundaries and may spontaneously regress [50]. It can be classified as linear or widespread [54]. Clinical approaches to scar treatment include surgical procedures, laser therapy, corticosteroid injections, and topical treatments like silicone gel. A scar gel based on HOCl has also been developed to improve scar appearance and alleviate associated itch and pain [53]. When applying HOCl in gel form, it is important that its structure closely resembles the natural HOCl produced by the human body [55]. HOCl gels and liquid products do not cause irritation, hypersensitivity, or toxicity. In a randomized, double-blind study, the efficacy, safety, and tolerability of HOCl scar gel were compared with 100% silicone gel in treating hypertrophic and keloid scars [6]. A multicenter trial over eight weeks measured the effects of HOCl gel (applied three times daily) or silicone gel in 44 adults with linear/widespread hypertrophic and keloid scars. When comparing HOCl scar gel with silicone gel, HOCl gel showed more favorable outcomes.

Although data on the effectiveness of HOCl scar gel remain limited, it has demonstrated certain advantages over silicone gel treatment [5,56]. Hypochlorous acid has proven effective and safe for postoperative care and for the prevention and treatment of hypertrophic and keloid scars [57]. It is available in solution, dermal spray, and scar gel forms. Due to its broad-spectrum antimicrobial and antibiofilm activity, HOCl solution reduces wound infection risk (compared to povidone-iodine) and promotes oxygenation at the wound site, which can accelerate healing [9,40]. The safety of HOCl solution is comparable to that of standard topical antiseptics [38]. HOCl scar gel has been shown to improve the appearance of hypertrophic and keloid scars and reduce associated itching and pain, and due to its safety and good tolerability, it is used alongside other treatments [38,51,53].

HOCl stands out with its efficacy and safety, especially when it is compared with other conventional antiseptics, for instance, sodium hypochlorite (NaOCl), povidone-iodine, or chlorhexidine [30,31,58]. HOCl has an ability to penetrate microbial biofilms and to inactivate bacteria, viruses, and fungi. What is interesting is that it is able to do this at much lower concentrations than NaOCl. Therefore, it minimizes tissue irritation and cytotoxicity to host cells. In contrast, widely used alternatives often require higher concentrations to achieve equivalent antimicrobial effects. However, this increases the risk of delayed healing or local adverse effects. Recent research also emphasizes the integration of computational and in silico methods to strengthen the comparative assessments of antiseptic agents. In silico studies are used to predict pharmacokinetic and toxicological profiles, as well as binding affinities to biological targets. This enables an early identification of compounds that combine strong antimicrobial activity with favorable safety margins. For instance, studies have demonstrated that in silico models facilitate screening of natural and synthetic bioactive agents to evaluate absorption, distribution, metabolism, and excretion (ADME)/Tox parameters, drug-likeness, and binding interactions. Thus, we are able to predict biological performance more accurately and cost-effectively than with wet-lab screening alone. Such approaches have confirmed HOCl’s favorable physicochemical characteristics (including solubility, rapid reactivity, and minimal systemic absorption), which support its clinical superiority over more aggressive antiseptics [58]. Furthermore, there is increasing interest in natural products as potential alternatives to synthetic chemical drugs, especially where issues of toxicity and long-term safety limit current therapies. Recent in silico and molecular docking studies have highlighted bioactive compounds from medicinal plants with promising pharmacological properties. These findings suggest that natural compounds can provide scaffolds for the development of novel therapeutics that combine efficacy with improved biocompatibility [59].

Research data from clinical studies on therapeutic use of HOCl and similar preparations use for ulcera/wounds are presented in Table 1 [38,41,42,43,44,55,60,61,62,63,64,65,66,67,68,69,70,71,72]. The majority of studies, 13 of them, have been conducted on patients with chronic wounds (including venous leg ulcers, diabetic foot ulcers, mixed-etiology ulcers, and other hard-to-heal lesions). These studies repeatedly show that HOCl reduces bacterial burden, improves wound-bed conditions, alleviates symptoms and often accelerates healing when used as an adjunct to standard wound care. However, only a very small number of studies have evaluated HOCl in the context of acute wounds, such as postoperative wounds, acute skin graft sites, or skin injuries in healthy volunteers. Although these few studies report beneficial effects, the evidence base for acute wound management remains limited.

## 4. Hypochlorous Acid in Infection Control and Topical Preparations for Dermatoses

The key therapeutic value of HOCl lies in its central microbicidal role (as a component of the innate immune response), where it acts through phagocytic cells (neutrophils, monocytes, and macrophages) [4,6,71,72,73,74]. HOCl’s antimicrobial mechanism differs from conventional antibiotics as it directly destroys microbial cells, including numerous Gram-positive and Gram-negative bacteria (such as *Staphylococcus aureus* and *Pseudomonas aeruginosa*), and their biofilms [5,71]. However, it is important to understand the balance of HOCl relative to other by-products formed after HOCl solution application. Its formulation, stability, pH, and resulting chemical reactivity affect the relative concentrations of HOCl compared to by-products like hypochlorite (-OCl), directly influencing antimicrobial activity, clinical efficacy, and potential irritation [3,72]. At a very acidic pH (pH < 3.5), HOCl concentration significantly decreases due to increased by-product production, while in alkaline environments (pH > 5.5), much of HOCl converts to -OCl. The stability of HOCl is optimized at a pH range of 3.5 to 5.5 [3,72,74]. Stabilized, pH-neutral HOCl demonstrates superior antimicrobial performance compared to unstable HOCl, including efficacy against strains resistant to -OCl [75,76,77].

**Figure 3:**
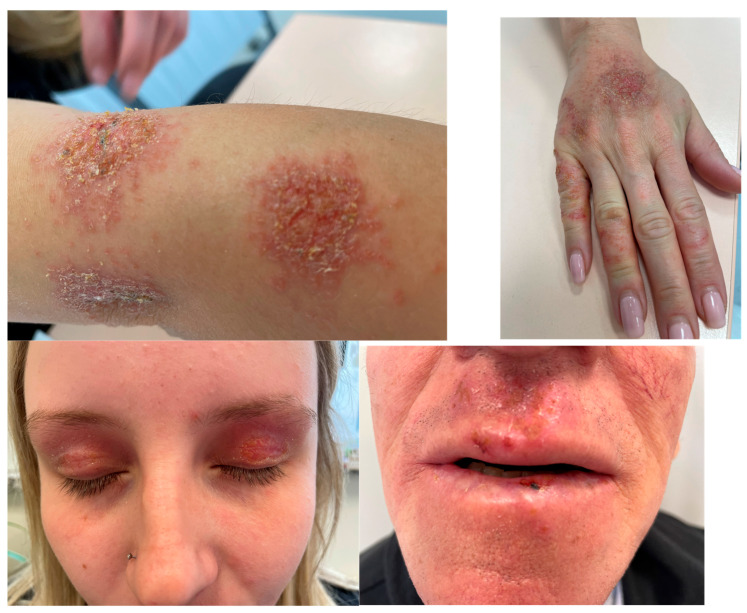
Examples of dermatoses that can be treated with HOCl: lesions such as those in atopic dermatitis (secondarily infected lesions) and contact dermatitis (two pictures above); as well as lesions near mucous membranes such as periocular skin lesions and perinasal/perioral eczema with secondary infection (two pictures below) (the figures were collected by the authors with consents of the patients).

Understanding the physicochemical properties of HOCl enabled the development of a stabilized, pH-neutral HOCl solution (Microcyn^®^ technology, Boulder, CO, USA), facilitating its clinical use [78]. Beyond its antimicrobial action, HOCl’s biological properties are also clinically significant in infammatory processes, as HOCl reduces the activity of inflammatory mediators involved in itch-related and inflammatory processes. Thus, HOCl can modify and inhibit the binding function of protein/molecules like IL-6 (by oxidizing specific amino acids), which may decrease the activity of other inflammatory/itch-related mediators like histamine and LTB4. HOCl directly modifies IL-6, including oxidation of methionine and tryptophan residues, which reduces its ability to bind to its receptor and activate downstream signaling (HOCl also reduces the activity of IL-2, which specific mechanism may differ from that of IL-6). In addition, during inflammation, activated immune cells (like neutrophils) produce HOCl, which can inactivate matrix metalloproteinases (MMPs) at the site of inflammation in tissues (e.g., the artery wall). Also, applying HOCl may helt wound healing by reducing excessive MMP activity, which is sometimes a predictor of poor healing, for example, in diabetic wounds. Thus, at high concentrations, HOCl also decreases the activity of MMPs such as MMP-7 and collagenase, inhibits mast cell degranulation and cytokine release, and positively influences keratinocyte function and fibroblast migration [9,79,80]. Therefore, research in dermatology indicates that HOCl exhibits antimicrobial, anti-inflammatory, and immunomodulatory properties advantageous in treating various skin disorders, including atopic dermatitis and other forms of eczema, seborrheic dermatitis, diabetic lower-extremity ulcers, pruritus, and acne vulgaris [9,10,81,82,83] (Figure 3). Application of HOCl to the skin of patients with atopic dermatitis reduces *Staphylococcus aureus* (which produces superantigens on atopic skin), potentially decreasing inflammation and benefiting treatment of eczematous dermatitis and itch relief in atopic dermatitis [84,85,86]. Clinical use of HOCl also includes promoting wound healing and preventing scar formation [40,41,42,43,44].

Overall, HOCl demonstrates a broad spectrum of antimicrobial activity (directly toxic to many bacteria and fungi, and potentially antiviral) and possesses anti-inflammatory and immunomodulatory properties confirmed in laboratory analyses. Topical application of HOCl shows therapeutic benefits for various skin diseases. Studies have shown that stabilized, pH-neutral HOCl formulations (including solutions, gels, and sprays) exhibit antimicrobial effects and are used in treating seborrheic dermatitis, itch associated with atopic dermatitis, acne vulgaris, diabetic foot ulcers, and hypertrophic and keloid scars. Clinical experience to date indicates that HOCl is well tolerated and considered safe, with no serious adverse effects reported.

Research data on HOCl and similar preparations for atopic dermatitis and acne are presented in Table 2 [14,84,85,87,88,89,90,91,92]. Most of these clinical studies reported similar findings, showing that bleach baths or sodium-hypochlorite cleansers improve clinical severity of atopic dermatitis. Five studies [conducted by Ryan et al. (2013) [87], Majewski et al. (2019) [84], Wong et al. (2013) [88], Huang et al. (2009) [90] and Stolarczyk et al. (2023) [92]] demonstrated clinical improvement, and two of them also showed a reduction in *Staphylococcus aureus* colonization or density, conducted by Majewski et al. (2019) [84] and Wong et al. (2013) [88]. However, two studies [conducted by Hon et al. (2016) [85] and Stolarczyk et al. (2023) [92]] did not confirm decreased in *S. aureus* colonization, and Asch et al. (2019) [89] found no reduction in systemic antibiotic use for AD superinfections. Most results support clinical improvement, but evidence for a consistent antimicrobial effect remains mixed. Evidence for the use of HOCl in acne treatment is limited, only one study conducted by Tirado-Sánchez et al. (2009) [14] found that a super-oxidized solution was as effective as benzoyl peroxide for inflammatory acne.

## 5. Hypochlorous Acid in the Prevention of Melanoma and Non-Melanoma Skin Cancers

Epidemiological associations between disinfection byproducts in drinking water and skin cancer risk stem largely from halogenated organic mixtures generated during large-scale chlorination, notably trihalomethanes and haloacetic acids. These species differ chemically and mechanistically from purified, stabilized HOCl formulations employed in dermatologic and antiseptic contexts; thus, risk signals from disinfection byproducts should not be conflated with the safety profile of controlled, topical HOCl use [93,94]. In experimental settings, exposure to HOCl has been shown to influence several interlinked pathways relevant to skin carcinogenesis. One key dimension is the modulation of inflammatory signaling. In ultraviolet (UV)-exposed epidermal models and murine skin, topical HOCl suppresses activation of stress-responsive transcriptional regulators such as NF-κB and AP-1, resulting in downstream diminution of inducible nitric oxide synthase (iNOS), cyclooxygenase-2 (COX-2), and other proinflammatory mediators. These alterations ameliorate UV-induced inflammation and, with repeated treatment, slow the progression of preneoplastic lesions to invasive non-melanoma tumors. In murine models predisposed to UV carcinogenesis, administration of HOCl reduced skin inflammatory gene expression and attenuated tumor formation, supporting a chemopreventive role mediated via inflammatory suppression [95].

Beyond direct suppression of inflammatory mediators, HOCl may exert paracrine antagonism of tumor-promoting NF-κB signaling through myeloperoxidase (MPO) derived from innate immune cells [23]. In melanoma models, endogenous HOCl produced by infiltrating myeloid cells has been implicated in trans-inhibition of IKK/NF-κB pathways within tumor cells, limiting tumor expansion. Knockout or pharmacologic inhibition of MPO accelerates melanoma growth, underscoring the significance of MPO-dependent HOCl flux in restraining tumor progression. Moreover, HOCl exposure may convert immunogenic proteins into forms more readily processed by antigen-presenting cells. Oxidative or chlorinative modification of tumor or viral antigens enhances uptake by dendritic cells and strengthens T-cell priming, thereby facilitating immune surveillance and antitumor responses. In vitro and ex vivo experiments demonstrate that HOCl-treated proteins are more efficiently internalized by dendritic cells and stimulate more robust adaptive immunity compared to untreated controls. In addition to immunomodulation, HOCl may act directly on transformed cells. A salient example is a HOCl-generating hydrogel (HOCl-CDS), which in murine melanoma models induced tumor cell cytotoxicity while remodeling the local immune microenvironment. When applied topically or peritumorally, this hydrogel slowed B16-F10 melanoma growth, prolonged survival, and potentiated immune checkpoint blockade efficacy [96]. This translational strategy illustrates how delivery of controlled HOCl fluxes can couple oxidative stress-induced cytotoxicity with enhanced antitumor immune responses.

Collectively, these preclinical findings support a conceptual framework in which HOCl acts on multiple fronts: reducing inflammatory drivers of carcinogenesis, moderating pro-tumor signaling within neoplastic cells, enhancing antigen presentation and immunogenicity, and directly inflicting oxidative damage on tumor cells. This multifaceted mechanism aligns HOCl with emerging paradigms in cancer prevention and adjunctive therapy [4]. It is important to emphasize that all present evidence derives from in vitro systems, organotypic human skin models, and animal models (e.g., SKH-1 high-risk cohorts, syngeneic murine melanoma). Clinical translation remains unrealized. Critical parameters such as optimal concentration, vehicle formulation, frequency and duration of application, exposure timing relative to UV insult, cutaneous penetration kinetics, and long-term safety in humans must be rigorously studied. Nonetheless, the accumulating mechanistic and experimental data establish HOCl as a compelling candidate for photochemoprevention of non-melanoma skin cancer, and as a potential adjunct in melanoma management [4,95].

## 6. Hypochlorous Acid in Dentistry

Dental care involves aerosol-generating procedures in close proximity to the oropharynx, where saliva-borne respiratory viruses can be present. Laboratory models simulating oral droplets/aerosols have shown that HOCl can achieve complete inactivation of enveloped viruses, including a SARS-CoV-2 (human betacoronavirus OC43), influenza A/H1N1, and HSV-1, within 30 s at 100 ppm under test conditions, with performance attenuated at lower concentrations and in the presence of saliva; parallel experiments reported inferior short-contact virucidal activity for hydrogen peroxide (H_2_O_2_) under the same conditions. These findings support HOCl as a candidate adjunct for reducing viral load in dental aerosols, while underscoring the influence of matrix effects and contact time on outcomes [13].

The stability and delivery of HOC1 in dental waterlines should also be mentioned here. Translating the previously mentioned results above to practice requires attention to the chemistry and handling of HOCl. Free available chlorine (FAC) exists as an acid–base pair (HOCl/OCl^−^); formulations that maintain a slightly acidic pH (~5–6.5) maximize the fraction of HOCl, the more potent, membrane-permeant species, and are the basis of “slightly acidic electrolyzed water” used in healthcare. Antimicrobial activity is strongly dependent on FAC concentration and pH, and decays with time, heat, light, and organic load. Accordingly, point-of-use generation or use of fresh, pH-controlled product is preferred over prolonged storage; monitoring of FAC (and pH) is recommended to ensure consistency. Public-health guidance documents describe superoxidized/HOCl solutions produced at pH 5.0–6.5 with high redox potential and emphasize that efficacy tracks FAC rather than nominal product label alone [97].

Within dental unit waterlines (DUWLs), biofilm and organic demand consume oxidants and can accelerate FAC loss. Bench and applied studies indicate that HOCl-based DUWL maintenance can reduce biofilm and heterotrophic plate counts, but fresh solutions perform better than material held in reservoirs for a week, and effectiveness depends on periodic “shock” disinfection combined with continuous low-level maintenance per manufacturer instructions. CDC recommends all dental units treat and monitor water to meet drinking-water standards (≤500 CFU/mL), independent of the specific agent used. Incorporating HOCl into DUWL protocols is feasible when paired with validated dosing, contact time, and routine microbiological monitoring [98,99].

Concerning HOCl safety and practicality, it should be mentioned that at concentrations around 100 ppm, HOCl solutions formulated at a mildly acidic pH are generally well tolerated on oral mucosa in short-contact applications, and several dental studies and guidance documents consider HOCl-based therapeutic water or mouthwash suitable adjuncts for reducing microbial load. With any oxidant, compatibility with materials (elastomers, metals) should be confirmed with unit manufacturers, and staff should monitor FAC and pH to avoid inadvertent under- or overdosing. Because organic load and flow-through dynamics in DUWLs may shorten effective contact time relative to bench models, clinics should verify water quality outcomes and reassess dosing as part of routine “Infection Prevention and Control” (IPC) quality assurance [13].

In summary, laboratory evidence supports rapid virucidal activity of HOCl at 100 ppm under simulated oral aerosol conditions. In practice, stable delivery requires controlling pH, using fresh or point-of-use-generated solution, accounting for DUWL biofilm demand, and embedding HOCl use within a broader aerosol mitigation strategy that addresses procedural and environmental variability [99]. Research data on the use of HOCl and similar preparations in dentistry are presented in Table 3 [12,98,100,101,102,103,104,105,106,107,108,109,110,111]. The majority of clinical studies showed very good effects of HOCl and similar preparations in dental practice. According to the results of clinical studies, HOCl and NaOCl-based products demonstrated consistent short-term antimicrobial effects, reducing total salivary bacterial counts and gingival inflammation. Several studies in periodontal disease, according to the results by Lin et al. in 2023 [12]; González et al. in 2015 [108]; Galván et al. in 2014 [110], reported significant reductions in bacterial load, plaque, or bleeding on probing. Mouthwash studies also showed that HOCl temporarily lowers oral bacterial levels (according to results by Lafaurie et al. in 2018 [98]), although the effect lacks long-term substantivity. In endodontics, increasing NaOCl concentration during root canal irrigation (according to the results by Ulin et al. (2020) [101] and Dahlstrand Rudin et al. (2024) [106] did not improve bacterial elimination or clinical outcomes [101,106]. In pediatric dentistry, NaOCl gel proved effective in caries removal and pulpotomy procedures [Alkhouli 2020; Karkoutly 2025; Chauhan 2017 [102,103,107]].

## 7. Hypochlorous Acid for Various Infections and COVID-19

Growing evidence shows that HOCl is very useful in treating various infections [112,113,114,115,116,117,118,119,120,121,122,123,124,125,126,127,128]. It is important to note that HOCl, as inorganic bactericidal compound of the innate immune system, is naturally produced as part of the cytotoxic system of myeloperoxidase in neutrophils [129]. When applied in vitro against various microorganisms, HOCl causes oxidation of nucleotides, inactivation of cells via enzymes, disruption of cell membranes, and rapid cell lysis. Solutions containing HOCl are extremely effective against all bacterial, viral, and fungal pathogens. According to in vitro studies, the antimicrobial activity of 0.01% HOCl surpasses that of other standard skin antiseptics, without showing cytotoxic effects, and is well tolerated even with long-term use and is not toxic to the ocular surface [130].

Beyond its established virucidal activity, HOCl also plays an important role in the prevention of respiratory viral transmission. As a fast-acting oxidant capable of inactivating SARS-CoV-2 on mucosal surfaces and environmental interfaces, HOCl supports the host’s first line of defense by reducing viral load before infection can occur. This concept aligns with broader prevention-focused strategies highlighted in recent COVID-19 literature, where interventions that strengthen early barrier immunity are emphasized as critical for reducing susceptibility to SARS-CoV-2. For example, a recent systematic review demonstrated that enhancing mucosal and innate immune responses can lower viral entry and transmission risk, underscoring prevention as a central component of pandemic control [131].

Due to its properties, HOCl represents an ideal adjuvant treatment for various infections including mucosal surfaces [123,132]. However, the presence of various organic compounds and inorganic ions accelerates the consumption of HOCl through oxidation reactions [16]. A significant advancement was achieved with the development of the patented Microcyn^®^ technology, which enables the generation of free chlorine without sodium hydroxide (NaOH) byproducts and has demonstrated broad-spectrum antimicrobial, anti-inflammatory, and immunomodulatory activity, as confirmed through numerous laboratory analyses.

Research data on the use of HOCl and similar preparations for infection prevention are presented in Table 4 [48,112,113,114,115,116,117,118,119,120,121,122]. The collection of studies demonstrates that HOCl and NaOCl preparations play a significant but context-dependent role in preventing infections. Several studies, including those by Yılmaz et al. (2025) [113] and Liu et al. (2017) [119], reported significant reductions in infection rates, showing antimicrobial effectiveness of HOCl-based solutions in ICU catheter care and intraoperative peritoneal lavage. Also, Alvarez et al. (2010) [116] and Macias et al. (2013) [117] showed that NaOCl performs comparably to povidone-iodine in reducing bacterial counts in short-term antiseptic applications. Fritz et al. (2011) [118] further supported the antimicrobial potential, bleach baths combined with mupirocin demonstrated the strongest reduction in *S. aureus* colonization. However, Kaplan et al. (2014) [115] observed that bleach baths with daily hygienic measures were not statistically significant in reducing of community-associated *Staphylococcus aureus* skin and soft tissue or invasive infections in children. Tran et al. (2021) [112] found that HOCl did not outperform chlorhexidine gluconate for skin antisepsis, emphasizing differences in antiseptic efficacy depending on the agent and context. Ciccia et al. (2018) [114] contributed safety data, showing that NaOCl was non-toxic for neonatal skin antisepsis. Some studies identified no benefit, such as Sevinç Gül et al. (2022) [120], which reported no reduction in SARS-CoV-2 viral load after HOCl gargling. Parcells et al. (2009) [48], demonstrated that NaOCl (Dakin’s solution) was less effective than imipenem irrigation in preventing postoperative infections after appendectomy. Panatto et al. (2022) [121], however, suggested that Sentinox HOCl spray may support faster viral clearance in mild COVID-19. Overall, multiple studies, including Yılmaz et al. (2025), Alvarez et al. (2010), Macias et al. (2013) [113,116,117], Fritz et al. (2011) [118], and Liu et al. (2017) [119], indicate consistent short-term antimicrobial benefits of HOCl/NaOCl solutions. At the same time, Tran et al. (2021) [112], Sevinç Gül (2022) [120], and Parcells et al. (2009) [48] show that these agents do not universally outperform other agents. Despite mixed results, HOCl and NaOCl preparations remain safe, well-tolerated, and effective in many clinical scenarios. The evidence suggests that combining antiseptics with additional preventive measures (such as hygiene education or intranasal therapy) may lead to better outcomes. In conclusion, the table demonstrates that HOCl and NaOCl have broad antimicrobial potential, but their real-world effectiveness varies.

## 8. Hypochlorous Acid in Ophthalmology and the Treatment of Eye Infections

Blepharitis is a common chronic disease of the ocular surface (affecting 37–47% of patients) that requires treatment by an ophthalmologist [122]. It occurs in older individuals and is often associated with additional ophthalmic complications that can worsen the condition and impede the healing process. It causes significant discomfort, with symptoms including redness, itching, burning, foreign body sensation in the eye, and blurred vision [122]. Bacteria may (but do not necessarily) be the primary cause of blepharitis, and the inflamed eyelid margins in affected patients are often colonized by bacteria. The main treatment approaches include topical and/or systemic antibiotic therapy (due to possible bacterial etiology) combined with topical steroids. Many patients with blepharitis require prolonged therapy due to the chronic nature of the disease and the temporary improvement of symptoms [123].

In November 2021, a group of Italian ophthalmologists participated in an online advisory board where they discussed the potential application of HOCl in the treatment of eye infections. Certain clinical experiences were presented, and adjuvant treatment with HOCl ophthalmic spray was proposed with the aim of shortening the clinical resolution time of blepharitis. Its potential benefit was particularly emphasized regarding ophthalmic complications that often accompany blepharitis [122]. According to the data, complications related to the eyelids may last several months [124]. Clinical cases suggest that adjuvant treatment with HOCl ophthalmic spray can reduce the duration of antibiotic and corticosteroid use, even in severe cases of blepharitis. This supportive therapy is backed by the anti-inflammatory properties of the stable HOCl formulation used in the ophthalmic spray, which not only reduces infections but also relieves itching, pain, and the risk of scarring, while helping to suppress damaging inflammatory responses.

The ophthalmic spray is indicated for periocular use but has proven to be safe and very well tolerated even in case of contact with the ocular surface [123,125]. Inflammatory lesions in the periocular region can be caused by various dermatoses, often involving changes in the bacterial microbiota of the skin. Gram-positive anaerobic cocci and skin commensals from the genus Corynebacterium stand out, which may contribute to the pathogenesis of periocular inflammatory skin diseases [126]. In therapy, prolonged application of HOCl ophthalmic spray can be useful in preventing recurrence of hordeolum. For example, untreated or poorly controlled blepharitis after cataract surgery in elderly patients can be a source of contamination and increase the risk of endophthalmitis. Prophylactic use of the ophthalmic spray may be an effective and safe solution for patients with recurrent or chronic blepharitis scheduled for cataract surgery. Furthermore, according to research, in patients with blepharitis, the application of 0.01% HOCl reduces the presence of residual bacterial species on the skin below the lower eyelid [127]. Cleansing the eyelids with HOCl does not affect the biological diversity of meibomian gland secretions in patients with internal hordeolum [128].

HOCl solutions have been developed and used in the production of commercial pharmaceutical formulations. In clinical practice, thanks to innovative technology, an electrolyzed ophthalmic spray based on pure, stable, and pH-neutral HOCl can be used as an antiseptic with high disinfection efficacy. The HOCl ophthalmic spray is a medical product that contains a 0.01% HOCl solution and represents the lowest concentration of pure HOCl approved for application on the ocular surface [133]. The use of HOCl in ophthalmic spray is indicated as an adjuvant in the treatment of blepharitis and for cleansing the periocular area before or after eye surgery [127]. Clinical use of HOCl ophthalmic spray has shown it to be an effective adjuvant for treating blepharitis and preventing related eye complications. In a case series of 10 patients, twice-daily HOCl spray over about 20 days led to symptom relief within days and full resolution of blepharitis. When combined with antibiotics, recovery was faster, with good tolerance and minimal side effects [123]. HOCl eye spray, in combination with antiviral therapy, is useful in treating herpes zoster infections. Two clinical cases of patients with herpes zoster were treated with the ophthalmic spray. Herpes zoster is a self-limiting disease caused by reactivation of the varicella-zoster virus (VZV), and the risk of VZV increases with age [134]. Reactivation occurs in cranial nerves (e.g., the trigeminal nerve), leading to herpes zoster ophthalmicus, whose symptoms usually resolve within 10–15 days following antiviral therapy [135]. Clinical experience in treating ocular herpes zoster has shown an advantage in combining antiviral therapy with HOCl ophthalmic spray, particularly in reducing symptoms and shortening treatment duration. Adjuvant treatment with HOCl resulted in shorter infection duration (compared to antiviral therapy alone) and prevention of excessive bacterial infection of skin lesions (often present in herpes zoster clinical presentation) [123].

In ophthalmology, the use of 0.01% HOCl offers numerous advantages over other antiseptic products for treating eye infections: immediate microbicidal action on all tested microorganisms compared to standard antiseptics (such as 70% isopropyl alcohol, 5% povidone-iodine, and 4% chlorhexidine gluconate), allowing application even to particularly sensitive and inflamed areas. Also, the HOCl-based eye spray formula is easy to handle, which promotes treatment continuity [136,137]. Overall, literature evidence and real-world clinical experience support the use of HOCl as an adjuvant in the treatment of eye infections and for periocular cleansing before and after eye surgery [123]. Research data on the use of HOCl and similar preparations in ophthalmology are presented in Table 5 [34,37,128,138]. The studies reviewed demonstrate that HOCl-based preparations can serve as treatment options for several ophthalmologic conditions. Two studies (Mencucci et al. in 2023 [132] and Zhang et al. in 2023) [34] indicate that HOCl is effective in the treatment of blepharitis, reducing bacterial load and improving ocular surface health [34,132]. Mencucci et al. (2023) [132] also reports additional benefits in patients with dry eye disease [132]. Yang et al. (2022) [128] shows that HOCl wipes do not change the microbial diversity of meibomian gland secretions in internal hordeolum [128]. Wang et al. (2023) [37] demonstrates that HOCl eye drops can accelerate healing in fungal keratitis without increasing complications, suggesting potential use in infectious keratitis [37]. Auclin et al. (2002) [138] suggests that HOCl antiseptic solutions can be used during cataract surgery preparation [138]. Overall, the available studies are limited in number, but their findings suggest that HOCl-based preparations hold therapeutic potential in ophthalmology.

## 9. Hypochlorous Acid in Otorhinolaryngology

Previous research has confirmed the effectiveness of NaOCl in treating various infections, but data on its application to the nasal mucosa (or perinasal skin) in patients with rhinitis or chronic rhinosinusitis is limited. Thus, the effects of NaOCl on cells may vary depending on tissue type [139]. It could be mentioned that NaOCl solutions generate HOCl/OCl^−^ in equilibrium and share the same reactive oxychlorine species responsible for antimicrobial activity; however, stabilized HOCl formulations used on skin and mucosa are typically pH-neutral or slightly acidic and are less irritating than classical alkaline NaOCl solutions. Raza et al. found that nasal irrigation with 0.05% NaOCl in saline safely and effectively relieved symptoms in patients with chronic *Staphylococcus aureus* rhinosinusitis, despite not fully clearing the bacteria [140]. It offered a low-cost, well-tolerated alternative to standard treatments, especially valuable in settings with limited access to antibiotics [140].

Because NaOCl and HOCl are closely related oxychlorine species, it is important to distinguish their formulations when discussing nasal applications. In aqueous solution, NaOCl dissociates to hypochlorite ions (OCl^−^), which exist in pH-dependent equilibrium with HOCl. At neutral or slightly acidic pH, HOCl predominates and is responsible for most of the broad-spectrum microbicidal activity, whereas at more alkaline pH, OCl^−^ is the dominant species and is generally more irritating and potentially more cytotoxic to mucosal epithelium. Stabilized medical HOCl preparations are therefore typically buffered to a neutral or slightly acidic pH, with lower irritancy compared to classical alkaline NaOCl (“bleach”) solutions. In the context of rhinology, currently available clinical data relate to low-concentration NaOCl nasal irrigation (0.05% in chronic *Staphylococcus aureus* rhinosinusitis), and we cite these studies as evidence that oxychlorine solutions can be used safely and effectively on the nasal mucosa. However, dedicated studies are still needed to evaluate intranasal use of pure, pH-neutral HOCl formulations, and any extrapolation from NaOCl data to HOCl should be made with this limitation in mind.

An in vitro study found that 0.05% NaOCl caused less cell damage than 0.5%, especially with a 5-min exposure compared to 15 min. Since nasal irrigation likely involves brief contact with the mucosa, around 5 min, 0.05% NaOCl was considered safer for potential in vivo use [140]. It was also shown that NaOCl cytotoxicity depends on its concentration and duration of exposure, as confirmed by the reduction in the F-actin network. This effect is believed to be mediated by interference with DNA or RNA synthesis [139]. In vitro tests on nasal epithelium showed that 0.05% NaOCl was far less toxic than 0.5%, especially with 5-min exposure twice daily. This concentration also caused fewer disruptions to cell structures like the F-actin network and ezrin, which are linked to tight junction integrity. Changes in these markers may signal altered cell polarization and potential tight junction opening [140].

Sinus lavage is also important, offering several advantages over topical or systemic antibiotics because it physically removes secretions and crusts from the nose, alters the mucus composition, and reduces the local concentration of bacteria, fungi, toxins, and proinflammatory substances released during the inflammatory response [141]. At the end of the third month of NaOCl treatment, objective measurements (rhinomanometry) showed a significant reduction in total nasal airway resistance. Active anterior rhinomanometry is a reliable method for assessing the functional status of the nasal cavity under various conditions and correlates well with subjective assessments of nasal resistance [142]. After treatment, despite improvements in the clinical picture, no significant changes in nasal nitric oxide (NO) production were observed [140].

Also, in patients receiving low-dose erythromycin for chronic rhinosinusitis over three months, despite substantial symptom relief, no significant increase in NO levels was detected. However, after 12 months, a trend of increasing NO levels was observed, likely due to gradual restoration of normal epithelial function, ciliary cell growth, and reduced inflammation [143].

Concerning variability in aerosol exposure and implications for HOCl use, data shows that real-world aerosol exposure is heterogeneous and reflects the procedure type (e.g., ultrasonic scaling, high-speed handpieces), device settings and flow, suction/HVE performance, room ventilation/air exchanges, and patient factors. Systematic reviews and professional guidance emphasize that preprocedural rinses and irrigation fluids can “reduce—but not eliminate” bioburden in generated aerosols, and that clinical efficacy depends on integrating multiple controls (HVE, rubber dams when feasible, ventilation/filtration) rather than relying on a single agent. Thus, if HOCl is used as a preprocedural rinse or as an in-line irrigant, protocols should specify concentration (e.g., 100 ppm), minimum contact time (≥30 s), and delivery mode (rinse vs. continuous irrigation), and should be implemented alongside engineering and administrative controls [99].

Finally, considering all that has been mentioned so far, the potential applications of HOCl in patient care are extensive, as confirmed by data from the literature. Research data on HOCl use in otorhinolaryngology are presented in Table 6 [140,144,145,146,147]. The majority of clinical studies showed beneficial effects of HOCl in field of otorhinolaryngology. Two studies, conducted by Cho et al. in 2016 [144] and by Yu et al. in 2017 [145], suggest that HOCl may offer symptom improvement in chronic rhinosinusitis, with Cho et al. [144] reporting better radiologic outcomes and Yu et al. [145] showing superior symptom relief compared to saline. In contrast, Jiang al. (2022) [146] found no additional benefit of HOCl over saline in post-FESS care, despite both improving endoscopic scores. Similarly, Kim et al. (2022) [147] demonstrated that HOCl effectively reduced symptoms in perennial allergic rhinitis but did not outperform saline. Overall, the evidence suggests that HOCl is beneficial, but current data do not consistently confirm its superiority over saline. 

## 10. Final Comments on HOCl Use in Clinical Practice

HOCl has a broad antimicrobial effect without inducing antibiotic resistance because it disrupts multiple cellular components, making it difficult for microorganisms to develop resistance. Importantly, HOCl is naturally produced in the human body, and when properly diluted, it is beneficial and non-toxic to the human body, and is generally well tolerated. It is primarily useful for wound healing and treating inflammatory conditions, as it promotes natural wound healing processes and is anti-inflammatory, making it useful for conditions like atopic dermatitis, acne, and eczema, among others.

In addition, HOCl is useful for mucosal surfaces because it has been confirmed that HOCl is safe for sensitive and mucosal areas (e.g., eyelids, nasal mucosa) and may also be used as a disinfectant (e.g., in healthcare settings). 

Generally, when looking/searching for topical therapy with antimicrobial, anti-inflammatory, and wound-healing properties, there are many “green” or herbal therapy options, including numerous plant-derived products (such as Aloe vera, Calendula officinalis, essential oils, and other botanical extracts) which have demonstrated antimicrobial, anti-inflammatory, and wound-healing properties, and several systematic reviews report positive effects in clinical wound care, sometimes comparable to standard agents like povidone-iodine [148,149,150]. However, these preparations often suffer from important limitations, including variable composition, lack of standardization, small and heterogeneous clinical trials, and the potential for contact dermatitis or sensitization, particularly with essential oils [151,152]. In contrast, HOC1 is an endogenous molecule with a well-characterized mechanism of action, rapid broad-spectrum activity against bacteria, fungi, and viruses, a very low propensity for resistance development, and an increasingly strong regulatory and clinical evidence base in wound care, ophthalmology, dentistry, and infection prevention [41,153,154,155]. Moreover, stabilized HOCl solutions or electrolyzed waters produced from salt and water degrade to simple saline and are increasingly discussed as “green” disinfectants with a favorable environmental profile when compared with many synthetic chemicals [156]. Taken together, current data do not support a complete replacement of HOCl by herbal or “green” remedies in high-risk clinical settings; instead, plant-based preparations may be considered complementary or adjunctive options in selected, lower-risk or cosmetic indications, provided that their quality, standardization, and safety are adequately controlled.

Although rare, negative effects of HOC1 can potentially include skin irritation, as its overuse may disrupt the natural condition of cutaneous tissue, leading to irritation and skin inflammation/redness. Also, with time, HOCl products may degrade and lose effectiveness, so it must be appropriately stored, i.e., in cool, dark places, and used within the recommended timeframe after opening. Also, since products with HOCl include skin sensitizers and/or preservatives, or substances at too high a concentration, they may potentially increase irritation risk. In addition, it must be mentioned that HOC1 is not a substitute for medical treatment (especially in severe or chronic dermatoses) and should not be expected to resolve underlying skin problems or provide long-lasting skin protection.

Despite its favorable safety profile compared with many traditional antiseptics, several limitations and safety considerations must be acknowledged when using HOCl, particularly in ophthalmic and dental applications. In ophthalmology, 0.01% HOCl is generally well tolerated and non-toxic to the ocular surface, even with repeated use; however, mild and transient stinging has been reported, and long-term safety data remain limited, especially for continuous daily use exceeding several months [123,130]. HOCl’s reactivity with organic matter may reduce its effective concentration on heavily soiled periocular skin, requiring repeated application to maintain antimicrobial efficacy [16]. In dentistry, short-contact use of mildly acidic HOCl mouthwash (≈100 ppm) is considered safe for oral mucosa, though higher concentrations or prolonged exposure may irritate soft tissues or interact with dental materials, underscoring the need for pH and free available chlorine monitoring during delivery through dental unit waterlines [11,12,98,100,101,102,103,104,105,106,107,108,109,110,111]. Importantly, HOCl’s rapid consumption by organic load and its lack of long-term substantivity mean that it cannot provide prolonged residual antimicrobial protection and must be applied repeatedly within clinical protocols [98]. These limitations highlight the importance of proper formulation, concentration control, and context-specific dosing to ensure both safety and therapeutic effectiveness.

From a regulatory and industrial perspective, stabilized HOCl formulations present several specific challenges. HOCl is inherently unstable and can gradually decompose into hypochlorite, chlorate and other chlorine species, with degradation strongly influenced by pH, temperature, light exposure, container materials and the presence of organic or inorganic impurities. This instability complicates the manufacture of bottled products that maintain a predictable concentration of free available chlorine and a high HOCl:OCl^−^ ratio over the labelled shelf life, particularly at higher concentrations [28,157]. Industrial processes therefore rely on tightly controlled electrolysis or acidification systems, in-line quality-control sensors and specialized packaging to limit loss of potency; patents and technical reports explicitly highlight product-to-product variability and the difficulty of achieving consistent stability of “stabilized HOCl” for at least several months under routine storage conditions [158,159]. These manufacturing and quality-control issues also underlie ongoing discussions in regulatory frameworks about how to define, test and label HOCl-based formulations, including the distinction between on-site generated electrolyzed water and pre-packaged medical products.

Despite the expanding body of research on HOCl, several important gaps and inconsistencies remain within the current literature. Much of the available evidence, particularly in wound care, ophthalmology and dentistry, relies on small, heterogeneous clinical studies that vary widely in formulation, concentration, pH, production method and duration of application, making direct comparison across studies challenging. Stabilized HOCl products differ substantially in their physicochemical properties, yet many reports do not fully describe formulation parameters such as free available chlorine, buffering systems, or shelf-life stability, which complicates reproducibility and limits the generalizability of clinical findings. In several therapeutic areas, including chronic wound management, periocular hygiene and oral health, long-term data are sparse, and many trials assess short-term microbiological endpoints rather than standardized clinical outcomes. Additionally, inconsistencies exist regarding HOCl’s efficacy in high-organic-load environments, where rapid consumption can reduce its antimicrobial activity, although not all studies quantify this effect. Finally, there is a notable lack of high-quality randomized controlled trials comparing HOCl directly with widely used antiseptics, as well as limited health-economic analyses assessing its cost-effectiveness across healthcare settings. Addressing these gaps will be essential to strengthen the evidence base and to guide the rational, standardized use of HOCl in clinical practice.

At the global policy level, HOCl has been the subject of recent applications to the WHO Model List of Essential Medicines for use in disinfection, antisepsis and wound care, which recognize its potential public-health impact but also emphasize the need for robust quality specifications, stability data and comparative clinical evidence before broad inclusion [30,31]. Against this background, several areas of future research are particularly important. First, well-designed in vivo safety studies are needed to characterize the effects of chronic or high-frequency exposure on the skin, ocular surface and oral mucosa, including in pediatric and other vulnerable populations. In parallel, long-term effectiveness data from pragmatic comparative trials against established antiseptics would help clarify HOCl’s clinical value across wound care, ophthalmology and dentistry. Additional work is also required to systematically profile the stability, degradation pathways and performance of industrially produced HOCl formulations under conditions that reflect real-world storage and use. Finally, formal health-economic and cost–benefit evaluations in diverse healthcare systems are essential, as preliminary reports suggest that stabilized HOCl solutions may provide comparable clinical outcomes at lower overall cost than many traditional antiseptic agents [160,161]. Generating these data would support more harmonized regulatory decisions and help clinicians and policymakers select HOCl products rationally within evidence-based guidelines.

## 11. Conclusions

Altogether, HOCl’s positive effects include combating microorganisms without stimulating resistance and promoting healing and anti-inflammatory effects, while potential negative effects are limited to irritation or disruption of the skin microclimate when applied too frequently/in high concentrations. Therefore, HOCl’s wide range of antimicrobial and anti-inflammatory effects are particularly useful for wound healing and treating various dermatoses. Excessive use, however, or improperly formulated products can cause skin irritation by disrupting the skin’s natural balance.

The application of HOCl and OCl^−^ (whether through immune responses or environmental factors) has been recognized as a beneficial therapy option which simultaneously acts as a key antimicrobial defense mechanism and induces various effects on human health. Particularly noteworthy is the role of HOCl in antimicrobial activity, regulation of skin inflammation, and photochemoprevention of skin tumor formation, highlighting its therapeutic potential. However, its effects must be carefully controlled to avoid unwanted damage. To fully harness the protective and therapeutic properties of HOCl, future advancements will rely on the development of new chemical compounds and sophisticated pharmaceutical formulations that enable precise regulation of chlorine-induced stress. Further research is essential to optimize HOCl dosing and to develop controlled-release systems aimed at maximizing both the anti-inflammatory and photoprotective effects of HOCl while minimizing tissue irritation and damage.

## Figures and Tables

**Table 1:** Research data on the use of HOCl and similar preparations for ulcers/wounds.

Author, Year	Participants (Number, Diagnosis)	Results/Conclusions
Valdová, V., 2025 [38]	237 patients with acute and chronic wounds	DebriEcaSan Alfa (neutral electrolyzed water solution) is a safe and effective adjunct in the treatment of acute/chronic wounds, demonstrating significant improvements in wound healing parameters.
Burian, E.A., 2022 [41]	20 healthy participants	Stabilized HOCl solution showed significant antimicrobial activity and accelerated wound healing, supporting its potential as an effective treatment for acute wounds.
Hiebert, J.M., 2016 [43]	17 adult patients with chronic open wounds	Ultrasound-assisted debridement with HOCl irrigation was more effective than saline in reducing bacterial regrowth in chronic open wounds.
Fazli, M.M., 2024 [44]	20 patients with chronic leg wounds in the first phase, 8 patients in the second pha	The SoftOx Biofilm Eradicator (composed of HOCl and acetic acid) was safe and well-tolerated by patients with chronic leg wounds. It showed promising, immediate antimicrobial effects and dose-dependent trends toward wound size reduction
Herruzo, R., 2023 [55]	220 patients with 346 chronic ulcers of various etiologies	Combined treatment with liquid and gel formulations of HOCl significantly improved healing outcomes in chronic ulcers and reduced the risk of infection.
Stough D., 2023 [60]	35 hair restoration surgery patients	Topical stabilized HOCl spray significantly reduced erythema and pruritus (with high patient compliance with treatment), and no tissue necrosis, supporting its valuableness in wound healing and postoperative care for hair restoration surgery.
Burian, E.A., 2023 [61]	12 split-skin graft transplantation patients	Stabilized HOCl in acetic buffer (HOCl + buffer) was well tolerated and showed promising wound healing, and had antimicrobial effects on, acute wounds, despite transient pain during treatment.
Selkon, J.B., 2006 [62]	30 patients with chronic venous leg ulcers	HOCl washes are an effective adjunctive therapy for chronic venous leg ulcers, enhancing healing and providing rapid pain relief.
Niezgoda, J.A., 2010 [63]	31 patients with venous or mixed venous/arterial leg ulcers	Vashe Wound Therapy significantly improved healing outcomes in patients with chronic leg ulcers, with 86% of lesions healed and a 47% reduction in size of non-healed wounds; it completely eliminated wound odor and resolved patient-reported pain.
Eliasson, B., 2021 [64]	57 patients with a lower extremity ulcer covered with devitalized tissue	Amino acid–buffered hypochlorite is effective and well-tolerated in treating hard-to-heal lower extremity ulcers. It reduces devitalized tissue, promoting wound healing and improving overall wound condition.
Serena, T.E., 2022 [65]	16 patients with hard-to-heal ulcers	Patients treated with NaOCl showed greater reductions in wound size and bacterial load compared to normal saline.
Lindfors J., 2004 [66]	11 patients with 18 wounds	Wounds treated with NaOCl cleanser consistently showed a reduction in bioburden and wound size compared to those treated with normal saline.
Assadian, O., 2018 [67]	260 patients with 299 chronic wounds	Wound irrigation with antiseptic solutions, particularly those containing hypochlorite/HOCl or agents like polihexanide, octenidine, or povidone-iodine, significantly reduced bacterial burden compared to saline.
Ricci, E., 2021 [68]	31 patients with hard-to-heal ulcers	Long-term use of HOCl oxidizing solution in combination with standard care was effective in promoting healing and improving wound conditions in patients with hard-to-heal ulcers.
Mohd, A.R., 2010 [69]	178 patients who underwent sternotomy	Dermacyn was safe and more effective as a wound-irrigation agent than povidone-iodine for preventing sternotomy wound infections.
Dalla Paola, L., 2006 [70]	218 patients with diabetic foot lesions	Super-oxidized solution is more effective and safer than povidone-iodine in treating infected diabetic foot lesions. It reduced bacterial load and accelerated healing time.
Martínez-De Jesús, F.R., 2007 [71]	45 patients with infected diabetic foot ulcerations	Neutral pH superoxidised aqueous solution is more effective and less toxic than conventional disinfectants in managing infected diabetic foot ulcers; it improves infection control, reduces odor and erythema, and promotes wound healing.
Piaggesi, A., 2010 [72]	40 patients with postsurgical infected diabetic foot lesions	Dermacyn^®^ Wound Care is more effective than povidone iodine in promoting healing of wide postsurgical lesions in infected diabetic foot.

Abbreviations: NaOCl—sodium hypochlorite; HOCl—hypochlorous acid.

**Table 2:** Research data on HOCl and similar preparations for atopic dermatitis and acne.

Author, Year	Participants (Number, Diagnosis)	Results/Conclusions
Majewski, S., 2019 [84]	50 children with moderate to severe AD and *S. aureus* skin colonization	Daily use of a 0.006% NaOCl body wash improved all outcome measures for *S. aureus* colonized AD in infants, children, and adolescents.
Hon, K.L., 2016 [85]	40 patients with moderate to severe AD	A regime of diluted bleach baths (4–2 times weekly) did not show superiority over water baths in terms of reducing *S. aureus* colonization or significantly improving AD severity.
Ryan, C., 2013 [87]	18 children with moderate to severe AD	Treatment with a cleansing body wash containing NaOCl led to a significant reduction in AD severity (scores IGA and BSA); the body wash was easier to use than traditional bleach baths.
Wong, S., 2013 [88]	36 patients (aged 2–30 years old) with moderate to severe AD	AD patients who took diluted bleach baths showed significant reductions in AD severity (EASI scores) and *Staphylococcus aureus* density, indicating clinical improvement.
Asch, S., 2019 [89]	753 children with AD	Diluted bleach baths or topical diluted acetic acid were not associated with decreased systemic antibiotic exposure for AD superinfections in the treatment of pediatric AD.
Huang, J.T., 2009 [90]	31 pediatric patients with moderate to severe AD and secondary bacterial infections	Chronic use of diluted bleach baths with intermittent intranasal application of mupirocin ointment decreased clinical AD severity in patients with clinical signs of secondary bacterial infections.
Stolarczyk, A., 2023 [92]	15 adults with AD and 5 healthy controls	Bleach baths improved AD severity, patient-reported pruritus, sleep quality, and physiological measures of skin barrier function in adults with AD, but they had no effect on qualitative or quantitative measures of cutaneous *S. aureus*.
Gonzalez, M.E., 2016 [93]	21 children with AD and 14 healthy controls	Both treatment groups (topical corticosteroids alone and corticosteroids plus bleach baths) saw significantly improved AD severity and restored microbial diversity on lesional skin, with no added clinical/microbiologic benefit observed from bleach baths.

Abbreviations: AD—atopic dermatitis; IGA—Investigator Global Assessment score; BSA—Body Surface Area; EASI—Eczema Area and Severity Index; NaOCl—sodium hypochlorite; HOCl—hypochlorous acid.

**Table 3:** Research data on the use of HOCl and similar preparations in dentistry.

Author, Year	Participants (Number, Diagnosis)	Results/Conclusions
Lin, Y.C., 2023 [12]	53 patients with periodontal disease, 30 healthy controls	100 ppm HOCl mouthwash significantly reduced total salivary bacterial count and the abundance of *Staphylococcus aureus* in patients with periodontal disease.
Lafaurie, G.I., 2018 [98]	75 healthy participants	HOCl mouthwash temporarily reduced bacterial counts in saliva but showed no lasting substantivity, with levels returning to baseline within one hour.
Zwicker, P., 2023 [100]	20 orally healthy participants	Both Granudacyn^®^ and Octenidol^®^ mouth rinses significantly reduced bacterial counts on the buccal mucosa and in saliva. Octenidine was more potent, but its cytotoxicity makes Granudacyn^®^ a safer option for patients with sensitive oral mucosa during cancer therapy.
Ulin, C., 2020 [101]	298 patients with endodontic diagnoses and root canal treatment	Increasing NaOCl concentration for irrigation during root canal preparation from 0.5% to 3% did not significantly affect the number of positive bacterial cultures or the frequency/intensity of postoperative pain. Patients treated with 3% NaOCl experienced a significantly higher postoperative swelling incidence.
Alkhouli, M.M., 2020 [102]	32 children with proximal caries of primary maxillary molars	CMCR agents like Brix 3000 and 2.25% NaOCl gel effectively removed carious dentine in primary teeth. Although conventional rotary treatment was faster, it caused significantly more pain.
Karkoutly, M., 2025 [103]	24 children with 48 carious first primary molars	The use of 2.25% NaOCl gel in pulpotomy of primary molars improved odontoblastic integrity, dentin bridge formation, and overall treatment outcomes without increasing adverse effects.
Iorio-Siciliano, V., 2021 [104]	40 untreated patients with severe/advanced periodontitis	Adjunctive use of NaOCl gel may enhance the effectiveness of MINST of periodontal pockets.
Bizzarro, S., 2016 [105]	110 patients with chronic periodontitis	Local disinfection with NaOCl, with or without systemic antimicrobials, did not provide additional long-term clinical or microbiological benefits compared to basic periodontal therapy alone.
Dahlstrand Rudin, A., 2024 [106]	298 patients who underwent root canal treatment	Root canal irrigation with either 0.5% or 3% NaOCl showed comparable long-term clinical outcomes after 5–7 years, with no significant differences in tooth survival, retreatment, or apical periodontitis.
Chauhan, S.P., 2017 [107]	40 children with carious primary molars who underwent pulpotomy	Both FC and 5% NaOCl showed high clinical success rates at 3 and 6 months, with slightly lower radiographic success for NaOCl; NaOCl is an effective alternative as a pulpotomy medicament for primary molars.
Gonzalez, S., 2015 [108]	12 patients with periodontitis	Twice-weekly oral rinsing with 0.25% NaOCl significantly reduced bleeding on probing, even in deep unscaled pockets (persistent gingival bleeding on probing was associated with a higher risk of periodontal breakdown).
De Nardo, R., 2012 [109]	40 healthy participants	0.05% NaOCl mouth rinse significantly reduced supragingival biofilm, gingival inflammation, and bleeding on probing (compared to water); extrinsic brown tooth staining was observed in all participants using NaOCl
Galván, M., 2014 [110]	30 patients with periodontitis	Twice-weekly rinsing with 0.25% NaOCl, compared with water, significantly reduced dental plaque and gingival bleeding
Coello-Gómez, A., 2018 [111]	20 patients with symmetrically impacted bilateral lower third molars	Both super-oxidized solution and 0.2% chlorhexidine gel mouthwashes enhanced postoperative recovery after lower third molar extractions.

Abbreviations: NaOCl—sodium hypochlorite; HOCl—hypochlorous acid; CMCR—chemo-mechanical caries removal; MINST—minimally invasive nonsurgical therapy; FC—formocresol.

**Table 4:** Research data on the use of HOCl and similar preparations for infection prevention.

Author, Year	Participants (Number, Diagnosis)	Results/Conclusions
Parcells, J.P., 2009 [48]	1063 patients who underwent appendectomy	Abdominal irrigation with an imipenem 1 mg/mL antibiotic solution significantly reduced postoperative surgical site infections compared to normal saline and Dakin’s solution (NaOCl).
Tran, A.Q., 2021 [112]	21 adults	Chlorhexidine gluconate as a skin antiseptic agent showed less bacterial growth, while HOCl, isopropyl alcohol, and povidone iodine showed no significant differences in bactericidal effects.
Yılmaz, Ş., 2025 [113]	60 patients in intensive care units	Catheter dressings with HOCl significantly reduced the incidence of central venous catheter-related bloodstream infections and signs of infection at the catheter exit site compared to povidone-iodine dressings.
Ciccia, M., 2018 [114]	105 infants who underwent central venous catheter placement	No evidence of skin toxicity in neonates undergoing central venous catheter placement with 0.05% NaOCl for skin antisepsis.
Kaplan, S.L., 2014 [115]	482 children treated for suspected *S. aureus* skin and soft tissue infection or invasive infection	Bleach baths combined with hygiene education showed a modest but non-significant reduction in recurrent MRSA skin and soft tissue infection compared to hygiene education alone, with no observed adverse effects.
Alvarez, J.A., 2010 [116]	48 healthy participants	Both 10% povidone-iodine and 10% NaOCl significantly reduced bacterial counts compared to baseline controls (no significant difference in antiseptic efficacy was observed between the two agents).
Macias, J.H., 2013 [117]	30 healthy participants	Chlorhexidine gluconate in isopropyl alcohol, NaOCl, and povidone-iodine showed comparable antiseptic efficacy for short-term procedures; only chlorhexidine showed a prolonged effect.
Fritz, S.A., 2011 [118]	300 patients with community-onset skin and soft-tissue infection and *S. aureus* colonization	Diluted bleach baths combined with intranasal mupirocin and hygiene education showed the highest and most sustained reduction in *S. aureus* colonization. Persistent recurrence of skin and soft-tissue infection highlights the need to address additional risk factors beyond *S. aureus* colonization.
Liu, J., 2017 [119]	212 patients with intestinal perforation who underwent opened surgical repair or partial intestinal resection	The use of Dermacyn for intraoperative peritoneal lavage effectively reduced the risk of infection due to its broad-spectrum bactericidal activity.
Sevinç Gül, S.N., 2022 [120]	75 patients hospitalized in the COVID-19 ward	After gargling with HOCl or povidone-iodine, no significant reduction in SARS-CoV-2 viral load was seen, which suggests that antiseptic mouth rinses may not play a meaningful role in preventing viral transmission.
Panatto, D., 2022 [121]	57 patients with mild COVID-19	Sentinox spray may accelerate viral clearance in mild COVID-19 patients (which demonstrated a broad virucidal spectrum) and was safe and well tolerated in both clinical and in vitro settings.

Abbreviations: COVID-19—coronavirus disease 2019; HOCl—hypochlorous acid; MRSA—Methicillin-resistant *Staphylococcus aureus*; SARS—Severe acute respiratory syndrome.

**Table 5:** Research data on the use of HOCl and similar preparations in ophthalmology.

Ophthalmology		
Zhang, H., 2023 [34]	67 patients with blepharitis	In patients with blepharitis topical 0.01% HOCl delivered via ultrasonic atomization is a safe and effective method for eyelid hygiene and provides improvements in ocular surface health and meibomian gland function.
Wang, H., 2023 [37]	96 patients with fungal keratitis	The use of HOCl solution in patients with blepharitis and dry eye disease causes significant improvements in tear film stability, ocular surface symptoms, and reduction in bacterial load, with outcomes superior to those observed with hyaluronic acid wipes.
Yang, S., 2022 [128]	8 patients with internal hordeolum	In patients with internal hordeolum, eyelid cleaning with HOCl wipes did not alter the overall biodiversity of meibomian gland secretions; the wipes reduced harmful bacterial pathogens while promoting beneficial symbiotic bacteria.
Mencucci, R., 2023 [132]	48 patients with blepharitis and mild to moderate dry eye disease	The use of HOCl solution in patients with blepharitis and dry eye disease causes significant improvements in tear film stability, ocular surface symptoms, and reduction in bacterial load, with outcomes superior to those observed with hyaluronic acid wipes.

Abbreviations: HOCl—hypochlorous acid.

**Table 6:** Research data on HOCl use in otorhinolaryngology.

Otorhinolaryngology		
Raza T., 2008 [140]	22 patients with persistent rhinosinusitis (*S. aureus* infection symptomatic carriers	0.05% NaOCl solution for nasal irrigation reduces symptoms, improves condition, and decreases airway resistance
Cho, H.J., 2016 [144]	37 children with rhinosinusitis	Both HOCl and isotonic saline nasal irrigation significantly improved total symptom scores in pediatric chronic rhinosinusitis. X-ray findings showed greater improvement in the HOCl group.
Yu, M.S., 2017 [145]	43 patients with chronic rhinosinusitis	Low-concentration HOCl nasal irrigation significantly improved chronic rhinosinusitis symptoms compared to saline irrigation (endoscopic scores and bacterial culture differences were not significant).
Jiang, R.S., 2022 [146]	78 FESS patients with chronic rhinosinusitis	HOCl nasal spray demonstrated similar effectiveness to normal saline nasal irrigation in post-FESS care, resulting in significant improvement in endoscopic scores.
Kim, H.C., 2022 [147]	139 patients with perennial allergic rhinitis	Low-concentration HOCl nasal irrigation significantly reduced allergic rhinitis symptoms (without causing adverse effects), but it did not provide additional symptom improvement compared to saline nasal irrigation.

Abbreviations: FESS—functional endoscopic sinus surgery; HOCl—hypochlorous acid.

## Data Availability

No new data were created or analyzed in this study.

## Abbreviations

The following abbreviations are used in this manuscript:

HOCl	hypochlorous acid
-OCl	hypochlorite
LTB4	leukotriene B4
IL-	interleukin
MMPs	matrix metalloproteinases
HSV-1	herpes simplex virus type 1
H_2_O_2_	hydrogen peroxide
TCID_50_	tissue culture infectious dose
VZV	varicella-zoster virus
NO	nasal nitric oxide
